# COVID-19 Knowledge, Risk Perception, and Precautionary Behavior Among Nigerians: A Moderated Mediation Approach

**DOI:** 10.3389/fpsyg.2020.566773

**Published:** 2020-11-20

**Authors:** Steven K. Iorfa, Iboro F. A. Ottu, Rotimi Oguntayo, Olusola Ayandele, Samson O. Kolawole, Joshua C. Gandi, Abdullahi L. Dangiwa, Peter O. Olapegba

**Affiliations:** ^1^Department of Psychology, University of Nigeria, Nsukka, Nigeria; ^2^Department of Psychology, University of Uyo, Uyo, Nigeria; ^3^Department of Psychology, University of Ilorin, Ilorin, Nigeria; ^4^Department of General Studie, The Polytechnic, Ibadan, Nigeria; ^5^Department of Psychology, Nigeria Police Academy, Kano, Nigeria; ^6^Department of Psychology, University of Jos, Jos, Nigeria; ^7^Department of Sociology, Federal University, Dutse, Nigeria; ^8^Department of Psychology, University of Ibadan, Ibadan, Nigeria

**Keywords:** COVID-19, knowledge, risk perception, precautionary behavior, Nigeria, gender

## Abstract

The novel coronavirus has not only brought along disruptions to daily socio-economic activities, but sickness and deaths due to its high contagion. With no widely acceptable pharmaceutical cure, the best form of prevention may be precautionary measures which will guide against infections and curb the spread of the disease. This study explored the relationship between COVID-19 knowledge, risk perception, and precautionary behavior among Nigerians. The study also sought to determine whether this relationship differed for men and women. A web-based cross-sectional design approach was used to recruit 1,554 participants (mean age = 27.43, SD = 9.75; 42.7% females) from all geopolitical zones in Nigeria, through social media platforms using a snowball sampling technique. Participants responded to web-based survey forms comprising demographic questions and adapted versions of the Ebola knowledge scale, SARS risk perception scale, and precautionary behavior scale. Moderated mediation analysis of the data showed that risk perception mediated the association between COVID-19 knowledge and precautionary behavior and this indirect effect was in turn moderated by gender. Results indicate that having adequate knowledge of COVID-19 was linked to higher involvement in precautionary behavior through risk perception for females but not for males. It was also noted that awareness campaigns and psychological intervention strategies on COVID-19 related activities may be particularly important for males more than females. Drawing from the health belief model, we recommend that COVID-19 awareness campaigns should target raising more awareness of the risks associated with the infection to make individuals engage more in precautionary behaviors.

## Introduction

The outbreak of the novel Coronavirus disease (COVID-19) pandemic has led to disruptions to health, economics, politics and social order all across the world. The COVID-19 is a ravaging and infectious viral disease caused by a novel strain of severe acute respiratory syndrome coronavirus 2 (SARS-CoV-2). Due to the rapidly increasing and contagious nature of the disease and its overwhelming influence on critical care and frontline healthcare staff as well as the possibility of transmission by asymptomatic carriers, governments around the world closed their borders, announced total or partial lockdowns, movement restrictions, social distancing, and wearing of facemasks (Biscayart et al., 2020; Zaka et al., 2020; Zhao et al., 2020) as precautionary measures to curb the spread of the virus. However, as of August 10, 2020, the total number of infected persons worldwide had risen to 19,718,030 cases with 728,013 deaths while Nigeria had 46,867 confirmed cases and 950 deaths (John Hopkins University, 2020). While the most developed regions of the world have battled with the virus, recording thousands of infections and deaths, it is unknown how Nigeria with a fragile and less sophisticated healthcare system will be able to confront the disease and stop it from spreading among its densely populated and already vulnerable populations. With no proven and acceptable pharmaceutical cure, the best way to curb the virus and prevent it from spreading, may therefore be the adoption of precautionary behaviors (Güner et al., 2020; Wilder-Smith and Freedman, 2020; World Health Organization WHO, 2020b). Precautionary behavior such as quarantine of infected persons, social distancing (e.g., self-isolation, school, workplace and market closures, cancelation of large public gatherings, etc.) and hygienic practices (e.g., frequent handwashing with soap, using a face masks, use of hand sanitizers, etc.) have been identified as infection control measures which help curtail the spread of infections (Leppin and Aro, 2009). In extreme cases, community-wide containments are also adopted (Sjödin et al., 2020). Hatchett et al. (2007) stated that the quarantine measure adopted during the 1918 Influenza pandemic was largely responsible for halting the widespread of the influenza. According to Li et al. (2004), the number of secondary cases from an infected patient during the SARS outbreak of 2003 was clearly reduced if the infected patient was isolated within 4 days after onset of symptoms. Also, de Vlas et al. (2009) asserted that the enforcement of community-wide containments in China, was instrumental in the consistent and great decrease of infections during the SARS epidemic. In Nigeria, Liberia and other parts of West Africa, hygienic practices widely adopted during the 2014–2016 Ebola outbreaks (Althaus et al., 2015; Czerniewska and White, 2020) and the 2012 Hepatitis E outbreak in South Sudan (Phillips et al., 2015) proved very useful in curbing the spread of infections across the regions. All across the globe, the most effective way of delaying the spread of infections, especially in times when vaccines are not yet available, has been the adoption of precautionary behaviors (Tang et al., 2012; Ebrahim and Memish, 2020; Sjödin et al., 2020). However, the challenge often is the knowledge and awareness level of individuals about the infectiousness of diseases (Hassan et al., 2017; Alam et al., 2020) and whether such knowledge will translate into precautionary behavior (Ho et al., 2013; Cain et al., 2018; Li et al., 2020).

Already, a growing number of health-related studies suggest that there is very little association between knowledge of an infectious disease and actual engagement in a precautionary behavior (see for review Phillips et al., 2015; Seimetz et al., 2016; De Buck et al., 2017). Studies now suggest that the path from knowledge/awareness to actual precautionary behavior is often mediated by certain factors such as risk perceptions/worry (Brug et al., 2004; Taglioni et al., 2013), self efficacy (Rimal, 2000) attention, information surveillance and elaboration (Raza et al., 2020), etc. Vartti et al. (2009) reported that people who are more knowledgeable about the related etiology of the disease, tend to worry more about being infected and therefore suggesting a link between knowledge and risk perception. It is worthy of note also that the trajectory of an infectious disease is often determined by the behavior of individuals, and the behavior is in turn related to individuals’ risk perception (Abdelrahman, 2020; Vijayaraghavan and Singhal, 2020; Zhang et al., 2020) and beliefs about the disease (Janz and Becker, 1984). Also, though there is relatively high knowledge of COVID-19 around the globe and among Nigerians in particular (Olapegba et al., 2020a,b), there is the possibility that competing myths and sacred narratives (Chukwuorji and Iorfa, 2020) may be downplaying involvement in precautionary behavior and therefore, it is important to investigate the path from knowledge/awareness of COVID-19 to precautionary behavior and the mediators and moderators that may be on this path. This will help in understanding the correlates and pathways to curbing the spread of the virus in Nigeria and other parts of the globe.

A study of this nature in the COVID-19 era is important because not only is a widely acceptable pharmaceutical cure currently unavailable, but amidst the high levels of social awareness of the pandemic coupled with its sudden and virulent nature, a lot of conspiracy theories have sprung up (Ahmed et al., 2020; Georgiou et al., 2020) leading to a possible decrease in willingness to engage in precautionary behaviors (Allington et al., 2020; Van Bavel et al., 2020). Perceptions about the nature and causes of the pandemic are also largely unfounded around evidence (Chukwuorji and Iorfa, 2020) and may influence engagement in precautionary behaviors among the people as well. Therefore, since issues related to precautionary health behavior in populations have been linked to individuals’ belief systems as well as their perceived fear or risks of contracting a disease, this study, borrowing from the health belief model (HBM) may offer explanations to the failure of the Nigerian people in adopting disease prevention strategies and screening tests for early detection and curbing the spread of the disease. Findings from this study may be used to guide health promotion and disease prevention programs in times of the COVID-19 or other pandemics and epidemics that may arise in the future.

The HBM has been used to explain and predict individual changes in health behaviors (Becker, 1974; Jones et al., 2015) and may also be important in understanding precautionary behaviors in the COVID-19 era. Specifically, the HBM offers risk (threat) perceptions (perception of susceptibility to a disease and perception of the severity of the disease) as important elements (channels of influence) that help in predicting individual health-related behaviors (Becker, 1974; Champion and Skinner, 2008; Cameron et al., 2009). This study therefore investigated how COVID-19 knowledge and risk perceptions could influence precautionary behaviors, specifically if risk perception may be mediating the relationship between COVID-19 knowledge and precautionary behaviors. Also, in line with Gustafson’s (1998) argument that gender structures give rise to systematic gender differences in the perception of risks, the study also sought to investigate if gender could moderate the mediating path from COVID-19 through risk perception to precautionary behavior. Although some studies have shown greater risk perception among males than females, the gender effect on risk behavior is partially mediated by risk propensity (a predisposition to risk) and this new and important insight tends to show that risk perception could assume any direction, depending on the potency of other underlying variables (Harris et al., 2006; Jayathilake, 2013). Moreover, some variables such as the greater perceived likelihood of negative outcomes have been found to partially mediate female’s lower propensity toward risk choices in gambling, recreation, and health domains (Harris et al., 2006) as well as the tendency of women being more risk averse than men (Nelson, 2015). Based on these considerations therefore, the following hypotheses were formulated:

• COVID-19 knowledge will predict precautionary behaviors such that higher levels of COVID-19 knowledge will give rise to higher levels of precautionary behavior.
• Risk perception will predict precautionary behaviors such that higher levels of risk perception will give rise to higher levels of precautionary behavior.
• Risk perception will mediate the prediction of precautionary behavior by COVID-19 knowledge (mediation hypothesis) and this effect will be stronger for females than their male counterparts (moderated mediation hypotheses).

**FIGURE 1:**
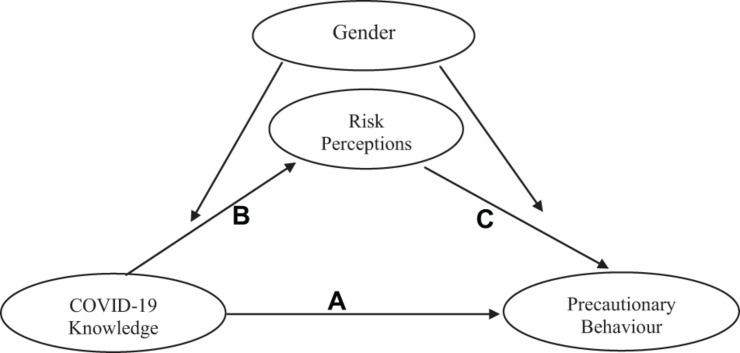
Conceptual model of moderated mediation for the effects of COVID-19 knowledge and risk perception on precautionary behavior.

The conceptual model of the expected moderated mediation is shown in Figure 1.

## Materials and Methods

### Participants and Procedure

This web-based cross-sectional study was conducted in Nigeria using self-administered questionnaires between 20 April and 30 April 2020. The general population of study covered participants aged 15 years and above in the 36 states of Nigeria and the Federal Capital Territory. Participants were recruited online through a blend of Respondent-Driven Sampling (RDS) technique and Random Survey Sampling (RSS). RDS was considered appropriate due to social restrictions associated with the COVID-19 lockdown (occasioned by the rapid spread of the disease) which made it difficult to physically access prospective respondents at the time of data collection. Some persons across States, regions, and local governments in Nigeria were considered, and therefore helped in disseminating the survey links to others in the same category who also responded to the questionnaires. RSS was considered appropriate because it enables the dissemination of an online semi-structured questionnaire, with a consent form attached to it. The link to the questionnaire was sent through social media platforms (such as WhatsApp, Facebook, Instagram, Telegram, and Nairaland) to the key persons. Prospective respondents were then driven to share the link of the survey to residents in other parts of the country. Persons with access to the Internet, who understood the English Language, and were as well willing to give informed consent, were included. To arrive at 1,554 responses, reminder messages with links to the Google form were posted online daily. Ethical approval was sought from the Faculty of Social Sciences Ethical Board, University of Ibadan. Strict adherence to the ethical provisions on confidentiality and autonomy was also observed. Informed consent was obtained from participants using an embedded form. Participants were informed of the confidentiality of the study and also informed they could withdraw from the study at any point if they so desired.

### Measures

The questionnaire assessed participants’ demographic characteristics (gender, age, marital status, ethnicity, educational qualification, religion, location, and perceived financial situation), and consisted of a battery of measures of COVID-19 knowledge, risk perceptions, and precautionary behavior.

#### COVID-19 Knowledge

COVID-19 knowledge was assessed using a 5-item Likert-type scale adapted from the Ebola knowledge scale (Rolison and Hanoch, 2015). Respondents’ knowledge of COVID-19 was arrived at by summing correct responses across item 1, source of COVID-19 [correct = (d)], item 2, transmission of COVID-19 [correct = (a), and (b),(c), or (d)], item 3, prevention of COVID-19, [correct = (b) and (d),(f), or (h)], item 4, symptoms of COVID-19 [correct = (a),(b), and (g)], and item 5, awareness of COVID-19 fatality, [correct = (a)], generating a maximum possible score of five.

#### COVID-19 Risk Perceptions

COVID-19 Risk Perceptions were measured using a 9-item Likert-type scale adapted from Brug et al. (2004)’s SARS risk perception Scale. Items were reworded to relate to coronavirus (e.g., “What level of threat do you think the COVID-19/Coronavirus pandemic poses to your job or studies?” and “How worried are you about contracting the Coronavirus?”). Participants rated these items on a 7-point Likert-type scale (1 = not at all likely, to 7 = extremely likely). A reliability coefficient (Cronbach’s alpha) of 0.71 was obtained in a pilot testing of the scale while the current data set yielded 0.75.

#### Precautionary Behavior

An adapted 10-item Likert-type scale was used to examine the precautionary behavior of participants during the COVID-19 pandemic (Barr et al., 2008; Duncan et al., 2009). The 10-item scale has statements dealing with actions taken in advance to protect against possible exposure to COVID-19. Sample items include: “I prefer to wash my hands pretty soon after shaking someone’s hand” and “I am comfortable going to very crowded places (reverse scored).” Participants rated items on a separate 7-point Likert type scale (one strongly disagree; seven strongly agree). Items four and six are reverse scored. A reliability coefficient (Cronbach’s alpha) of 0.80 was obtained in a pilot study of the scale while the current data set yielded an alpha of 0.75.

### Data Analysis

The data were analyzed using SPSS version 22.0 software. Descriptive statistics, using frequencies, percentages, means, and standard deviations, were conducted for socio-demographic variables and precautionary behavior. To compute COVID-19 knowledge, the mean score and standard deviation for the sample population were calculated and scores above the norm were indicative of high knowledge of COVID-19, while scores below the norm indicated low knowledge of COVID-19. For risk perception and precautionary behavior, scores in each scale were summed to obtain a composite aggregate for each measure. Higher scores indicated higher risk perception or precautionary behavior (as the case was). Pearson’s correlation was used to establish the relationship between the demographics and major variables of interest. Moderated mediation analysis was carried out with model 58 of Hayes PROCESS macro for SPSS (Hayes, 2018). The model 58 of the Hayes PROCESS macro uses ordinary least squares (OSL) analysis for calculating the mediation and the moderated mediation effects and bootstrapping for calculating the confidence intervals (CI) (Miers et al., 2017). Our choice of these statistics was influenced by the consideration that a moderated mediation would test the influence of a fourth variable (gender) on the mediated relationship between COVID-19 knowledge and precautionary behavior. In this moderated mediation model, precautionary behavior was entered as the outcome variable, COVID-19 knowledge as the independent variable, risk perception as the mediator. Gender was included as the moderator on the dependent variables risk perception and precautionary behavior. Age was included as a covariate in the model. See Figure 1 for a representation of the conceptual model.

## Results

Participants were 1,554 persons (42.7% females and 57.3% males; mean age = 27.43, SD = 9.75). The sample characteristics of participants, including educational qualification, perceived financial status, relationship status, religion, and geopolitical groupings, and their statistical values are shown in Table 1.

**TABLE 1:** Sample characteristics (*n* = 1,554).

Variables	Categories	Frequency	Percent
Gender	Female	664	42.7
	Male	890	57.3
Educational qualification	High school	399	25.7
	Diploma	343	22
	Degree	471	30.3
	Higher degree	301	19.4
	Professional	40	2.6
Perceived financial status	Don’t meet basic needs	288	18.5
	Just meet basic needs	536	34.5
	Meet needs with a little left	440	28.3
	Live comfortably	290	18.7
Relationship status	Single/not dating	618	39.8
	Single/but dating	578	37.2
	Married	350	22.5
	Others	8	0.5
Religion	Christianity	989	63.6
	Islam	555	35.7
	Others	10	0.7
Geopolitical zones	North Central	231	14.9
	North West	141	9.1
	North East	22	1.4
	South East	66	4.1
	South South	60	3.9
	South West	1,034	61.6

Means, standard deviations, and correlations among the observed variables were computed separately for males and females and shown in Table 2. Among females, being older (older age) was related to higher COVID-19 knowledge (*r* = 0.09, *p* < 0.05), and higher precautionary behavior (*r* = 0.10, *p* < 0.01), but not risk perception (*r* = 0.04, *p* > 0.05). Higher COVID-19 knowledge was related to greater risk perception (*r* = 0.09, *p* < 0.05) and greater precautionary behavior (*r* = 0.18, *p* < 0.01). Higher risk perception was related to greater precautionary behavior (*r* = 0.23, *p* < 0.01) (Table 2).

**TABLE 2:** Means, standard deviations, and intercorrelations of study variables separated by gender (*n* = 1,551).

Variables	1	2	3	4
Age	–	0.09*	0.04	0.10**
COVID-19 knowledge	0.10**	–	0.09*	0.18**
Risk perception	0.10**	0.06	–	0.23**
Precautionary behavior	0.10**	0.18**	0.25**	–
Females (*n = 664)*				
Mean	25.52	3.71	35.69	56.90
SD	8.22	0.82	10.41	10.69
Males (*n = 890)*				
Mean	28.85	3.71	35.94	55.80
SD	10.53	0.83	10.66	10.62

*^∗∗^*p* < 0.01; ^∗^*p* < 0.05; Correlations for females are above the diagonal while correlations for males are below the diagonal.*

For males, being older (older age) was related to higher COVID-19 knowledge (*r* = 0.10, *p* < 0.01), higher risk perception (*r* = 0.10, *p* < 0.01), and higher precautionary behavior (*r* = 0.10, *p* < 0.01). Higher COVID-19 knowledge was related to greater risk perception (*r* = 0.250, *p* < 0.01) and higher precautionary behavior (*r* = 0.18, *p* < 0.01). Risk perception was not related significantly to precautionary behavior (*r* = 0.06, *p* > 0.05).

In Table 3 it was found that older age predicted increased risk perception. Gender did not predict risk perception. Greater COVID-19 knowledge predicted elevated levels of risk perception. Gender did not moderate the association of COVID-19 knowledge and risk perception, given that the interaction term was not significant. The predictors accounted for 1% of the variance in risk perception [R^2^ = 0.01, *F*(4,1549) = 4.07, *p* = 0.003].

**TABLE 3:** Moderated mediation results for the link between COVID-19 knowledge and precautionary behavior.

Predictors	β	SE	*t*	*p*	95% CI
Outcome: risk perception					
Age	0.08	0.03	2.92	0.00	(0.03, 0.14)
Gender	−0.03	0.55	−0.05	0.96	(−1.10, 1.05)
COVID-19 Knowledge (C-19 K)	0.78	0.33	2.41	0.02	(0.14, 1.42)
C-19 K*Gender	−0.39	0.66	−0.60	0.55	(−1.68, 0.89)
Outcome: precautionary Behavior					
Age	0.08	0.03	2.82	0.01	(0.02, 0.13)
Gender	−1.42	0.53	−2.68	0.01	(−5.4, 1.83)
COVID-19 Knowledge (C-19 K)	2.04	0.32	6.47	0.00	(1.42, 2.66)
Risk perception	0.23	0.03	9.25	0.00	(0.18, 0.28)
Risk perception*Gender	0.01	0.05	2.82	0.83	(−0.09, 0.11)
Conditional indirect effects					
Female	0.22	0.12			(0.01, 0.50)
Male	0.14	0.11			(−0.05.37)

**FIGURE 2:**
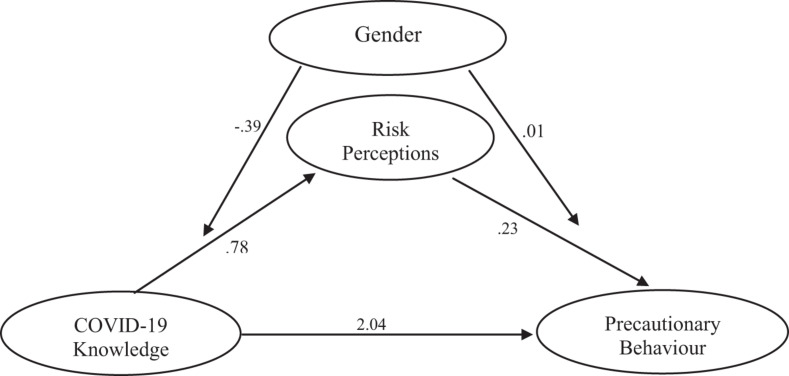
Moderated mediation model showing standardized coefficients.

In Table 3 also, it was found that older age predicted increased precautionary behavior. Gender did not predict precautionary behavior. Greater COVID-19 knowledge predicted elevated levels of precautionary behavior. Higher levels of risk perception predicted higher levels of involvement in precautionary behaviors. Gender did not moderate the association of risk perception and precautionary behavior, given that the interaction term was not significant. Our hypothesis of a moderated mediation effect was supported as evidenced by a significant indirect effect of COVID-19 knowledge on precautionary behavior through risk perception among females [B = 0.22, 95% CI = (0.01, 0.50)], but not males (B = 0.14, 95% CI = −0.05, 0.37). Note that the moderated mediation is significant when the 95% CI did not encompass zero as shown in the case for females. The predictors accounted for 9% of the variance in precautionary behavior [R^2^ = 0.09, *F*(5,1548) = 31.46, *p* = 0.00]. The moderated mediation model with standardized coefficients is presented in Figure 2.

## Discussion

The study investigated COVID-19 knowledge, risk perception, and precautionary behavior among Nigerians and the moderated-mediation effect among the variables. Findings showed that COVID-19 knowledge had a significant influence on precautionary behavior. This supported our hypothesis and mirrored previous findings (Brug et al., 2004; Li et al., 2020). It is logical to expect that when individuals are aware of threats, they will adopt reasonable behaviors that may avert the threat from causing harm. However, other studies (e.g., Phillips et al., 2015; Seimetz et al., 2016; De Buck et al., 2017) did not report this relationship but rather suggested that knowledge in itself may not always lead to precautionary health behaviors until the relationship is mediated upon by other factors (Rimal, 2000; Taglioni et al., 2013; Raza et al., 2020). These may on the other hand explain the observation that even though the knowledge of the pandemic is relatively high, some individuals still do not adhere to precautionary measures according to the protocol laid down by authorities [see Table 4 (Appendix Table 1)]. They may be said to be exhibiting attitude-behavior discrepancies, a tendency to be oblivious of the negative consequences due to inherent self-interests (as can be seen in pockets of disobedience to precautionary health behaviors in the face of the pandemic).

Consistent with previous findings (e.g., Abdelrahman, 2020; Vijayaraghavan and Singhal, 2020; Zhang et al., 2020) therefore, we postulated also that perception of risk will serve as a pathway through which knowledge and awareness of COVID-19 will influence precautionary behavior and that this influence may be more for females than for males. We also tested the direct influences of risk perception, age, and gender on precautionary behavior. As hypothesized, risk perception significantly predicted precautionary behavior, agreeing with previous findings (Brug et al., 2004; Lau et al., 2010). This implies that perception of risk is an important variable that could inform valid and reliable precautionary behaviors and possible means of preventing a newly emerging contagious disease like COVID-19. Studies supporting the present result suggested that an individual’s ability to promote precautionary behavior largely relies on the perceived risk of contracting a disease and that risk perception is a strong predictor of precautionary behaviors (Lau et al., 2010; Zhang et al., 2020). Moreover, as outlined by the HBM, for individuals to willingly engage in precautionary behaviors, they may have to, first and foremost, significantly perceive the risk that such disease poses to them.

The study also showed that being older predicted more precautionary behavior and this is supported by Zhang et al. (2020)’s study. This finding indicates that older people and those with underlying comorbid diseases took more precautionary measures compared with younger people. International and national medical agencies have stated that older people and those with underlying diseases are more vulnerable to coronavirus and at risk of dying (World Health Organization WHO, 2020b). This may explain why elderly respondents reported engaging more in precautionary measures compared to the younger respondents who engaged in less precautionary behaviors as observed in the study.

The result that gender differences existed in precautionary behavior is in line with past studies where females have consistently been found to engage in more precautionary behaviors than their male counterparts (Brug et al., 2004; Bish and Michie, 2010). This implies that females have a greater tendency than males to engage in precautionary behaviors such as the washing of hands, use of hand sanitizers, wearing of face masks, cleaning of surfaces, and having plans to visit a hospital or call emergency numbers in case of suspected symptoms, etc. This may be related to the perceived global vulnerability of females to illness (Duncan et al., 2009; Dellar et al., 2015). This relationship between gender and precautionary behavior may probably indicate that females perceive themselves as more susceptible to adverse conditions than males.

Also, it was observed that risk perception mediated the relationship between COVID-19 knowledge and precautionary behavior. This outcome relates to previous studies, for instance, a study conducted among the Saudi and non-Saudi Arabian pilgrims in 2014 on the outbreak of Middle East respiratory syndrome showed that overall knowledge of causative agents, the symptoms of the virus and its similarity to the disease and risk perception of the virus were associated with precautionary behavior (Hosam et al., 2017). The results showed that knowledge influenced precautionary behavior through the perception of risk. In other words, individuals who reported high levels of knowledge of the disease but did not perceive it as a risk did not initiate and engage in precautionary behaviors. This finding also strongly supports the assertion of the HBM that risk perception serves as a channel of influence that helps in predicting health behavior (Becker, 1974; Champion and Skinner, 2008; Cameron et al., 2009).

We hypothesized that the effect of the mediator (risk perception) will be moderated by another variable (gender). This hypothesis was influenced by previous findings that risk perception differs across gender. Most times the moderation may occur on any or all path(s) in the mediation model. Although, few studies have investigated if gender moderates the pathway of knowledge/awareness of an infectious disease through risk perception to precautionary health behavior, our study contributed to existing body of knowledge on the moderating influence of gender on the pathway of knowledge/awareness of an infectious disease through risk perception to precautionary health behavior, thus advancing the science in this area for other researchers to explore. Though no direct study with similar focus and results were found, a meta-analytic study on gender differences in risk perception pointed out that gender influences perception of risk (Byrnes et al., 1999). Also, owing to the offspring risk hypothesis (Harris et al., 2006), females tend to perceive greater risks than males because they are primary caregivers by nature and if people perceive more risks in the world, they will possibly be more effective at preserving any offspring under their care. Moreover, this finding corroborates a recent study that showed gender differences in risk perception of drug use between males and females (Ottu and Umoren, in press). In sum, our findings showed that risk perception serves as a pathway through which COVID-19 knowledge may influence precautionary behavior and that this may be higher in females than in males.

### Implication of Findings

Much more efforts are needed to understand the factors that promote precautionary behaviors in times of pandemics and these efforts may benefit from the findings in this study. One, the dynamic nature of infectious disease transmission suggests that behavior by several individuals may have a significant impact on the trajectory of an outbreak. However, individuals may not take precautions if they are not aware or have wrong or inadequate knowledge about the outbreak. Therefore, in line with the findings of our study that more accurate COVID-19 knowledge predicted greater precautionary behavior, and coupled with the fact that there are already myths and conspiracy theories surrounding the origin and nature of COVID-19 (Ahmed et al., 2020; [Bibr B22][Bibr B49]), we recommend enlightenment campaigns aimed at promoting adequate knowledge of the COVID-19 pandemic as well as addressing the current misconceptions and misinformation about the disease. In places where such knowledge is already influenced by conspiracy theories, it is important to reorient the public on the real nature and origin of the disease. In unaffected areas, true risks may be low, but due to the worldwide media coverage of the pandemic, there is the possibility of elevated levels of risk perception. Therefore, the scientific community may leverage on this to explore ways to best communicate risks to individuals without unnecessarily causing panic. Individuals who perceive themselves as being at risk of contracting the virus should engage in precautionary behaviors as suggested in this study rather than form stereotypes and prejudices against persons perceived to be the sources of the disease outbreak as reported in earlier findings (Chukwuorji and Iorfa, 2020; Olapegba et al., 2020a,b). It is therefore, necessary that knowledge, realistic risks, and effective precautionary behaviors are communicated through various information sources so that people can engage in more preventive behaviors than depend on vaccines.

Consequently and in line with the HBM, individuals may need to be informed about the potential risks of infection to adopt the right precautionary measures (Brug et al., 2004). Campaigns at raising awareness of risks should therefore convey the consequences of the health issues associated with risk behaviors in a clear and unambiguous fashion. This will help individuals to understand perceived severity of the risks associated with the disease. Measures to control future outbreaks should not be limited to the development of vaccines, but also adequately informing the public about the true nature (and origin of the infections) as well as risks as these have shown to be predictors of precautionary behavior. The findings from this study along with previous findings justify the need to look more closely at the drivers of precautionary behaviors in individuals. This will serve as a proactive step toward curbing future endemics or pandemics.

## Conclusion

Our study revealed, COVID-19 knowledge predicted precautionary behavior and risk perception significantly predicted precautionary behavior. Moreover, age and gender emerged as important variables associated with precautionary behavior. Older people readily showed a heightened tendency toward precautionary behavior than young persons. Females had higher likelihood of exhibiting precautionary behavior compared to males. In the area of mediation, risk perception aided the relationship between COVID-19 Knowledge and Precautionary behavior. In other words, knowledge influenced precautionary behavior through the perception of risk. The test for moderation indicated that gender (in this case, being female) was important as a precautionary index in risk perception than being male. Specifically, our results show that gender moderated the indirect path from COVID-19 Knowledge to precautionary behavior. It was also noted that awareness campaigns and psychological intervention strategies on COVID-19 related activities may be particularly important for males than females. Drawing from the health belief model, we recommend that COVID-19 awareness campaigns should target raising more awareness of the risks associated with the infection to make individuals engage more in precautionary behaviors.

### Limitation of the Study

This study relatively utilized a limited number of participants considering the population of the country; studies utilizing larger sample sizes are suggested. The use of an online web survey data collection approach in our study may have resulted in underrepresentation of the unprivileged and the uneducated. The implication is that the views of that section of the society were left out of the study. This raises the issue of coverage in addition to other methodological concerns associated with non-probability sampling and these could pose problems of generalization. Thus, our findings should be treated with caution without considering these as representative of adequately sampled population with optimal distribution across commonly described socioeconomic strata. To our knowledge, this is the first study on the relationship between COVID-19 knowledge, risk perception, and precautionary behavior among Nigerians. This data could be used as a baseline to explore differences with samples made up of persons without access to the Internet.

## Data Availability Statement

The raw data supporting the conclusions of this article will be made available by the authors, without undue reservation.

## Ethics Statement

Ethical review and approval was not required for the study on human participants in accordance with the local legislation and institutional requirements. The patients/participants provided their written informed consent to participate in this study.

## Author Contributions

SI, OA, and PO designed the study. OA, RO, SK, JG, AD, SI, IO, and PO contributed to perform the investigation and data collection. SI and OA analyzed the data. SI, RO, and SK drafted the initial manuscript. SI, IO, OA, and PO critically revised the manuscript for important intellectual content. All authors discussed the results and contributed to the final manuscript.

## Conflict of Interest

The authors declare that the research was conducted in the absence of any commercial or financial relationships that could be construed as a potential conflict of interest.

## Appendix

**TABLE 4:** Precautionary behavior engaged in by Nigerians.

Precautionary behavior		Disagreement	Undecided	Agreement
It really bothers me when people sneeze/cough without covering their mouths	Females	69 (10.4)	40 (6.0)	555 (83.6)
	Males	74 (8.3)	83 (9.3)	733 (82.3)
	Total	143 (9.2)	123 (7.9)	1,288 (82.9)
I wash/sanitize my hands pretty soon after shaking someone’s hand	Females	79 (11.9)	44 (6.6)	541 (81.5)
	Males	100 (11.2)	81 (9.1)	709 (79.7)
	Total	179 (11.5)	125 (8.0)	1,250 (80.4)
I avoid touching structures like door handles and stair case railing at public places	Females	94 (14.2)	50 (7.5)	520 (78.3)
	Males	143 (16.1)	84 (9.4)	663 (74.5)
	Total	237 (15.3)	134 (8.6)	1,183 (76.1)
I avoid wearing face mask	Females	345 (52.0)	88 (13.3)	231 (34.8)
	Males	473 (53.1)	125 (14.0)	292 (32.8)
	Total	818 (52.6)	213 (13.7)	523 (33.7)
I want people to be tested for coronavirus	Females	146 (22.0)	71 (10.7)	447 (67.3)
	Males	181 (20.3)	96 (10.8)	613 (68.9)
	Total	327 (21.0)	167 (10.7)	1,060 (68.2)
I don’t mind going to very crowded places	Females	555 (83.6)	31 (4.7)	78 (11.7)
	Males	727 (81.7)	60 (6.7)	103 (11.6)
	Total	1,282 (82.5)	91 (5.9)	181 (11.6)
I will self-isolate myself at home if needed	Females	74 (11.2)	37 (5.6)	553 (83.3)
	Males	94 (10.6)	40 (4.5)	756 (84.9)
	Total	168 (10.8)	77 (5.0)	1,309 (84.2)
I frequently sanitize/wash my hands with soap and water	Females	104 (15.7)	51 (7.7)	509 (76.7)
	Males	176 (19.8)	112 (12.6)	602 (67.6)
	Total	280 (18.0)	163 (10.5)	1,111 (71.5)
I avoid going to public places	Females	78 (11.8)	39 (5.9)	547 (82.4)
	Males	115 (12.9)	95 (10.7)	680 (76.4)
	Total	193 (12.4)	134 (8.6)	1,227 (79.0)
I have changed my lifestyle because of coronavirus	Females	113 (17.0)	38 (5.7)	513 (77.3)
	Males	139 (15.6)	72 (8.1)	679 (76.3)
	Total	252 (16.2)	110 (7.1)	1,192 (76.7)
